# Spinal Astrocytic Activation Is Involved in a Virally-Induced Rat Model of Neuropathic Pain

**DOI:** 10.1371/journal.pone.0023059

**Published:** 2011-09-28

**Authors:** Gui-He Zhang, Miao-Miao Lv, Shuang Wang, Lei Chen, Nian-Song Qian, Yu Tang, Xu-Dong Zhang, Peng-Cheng Ren, Chang-Jun Gao, Xu-De Sun, Li-Xian Xu

**Affiliations:** 1 Department of Anesthesiology, Tangdu Hospital, The Fourth Military Medical University, Xi'an, People's Republic of China; 2 Department of Anesthesiology, School of Stomatology, The Fourth Military Medical University, Xi'an, People's Republic of China; 3 Central Laboratory, The Fourth Military Medical University, Xi'an, People's Republic of China; 4 Department of Gynecology and Obstetrics, Naval General Hospital, Beijing, People's Republic of China; 5 Department of Hepatobiliary Surgery, PLA General Hospital, Beijing, People's Republic of China; 6 Department of Ultrasound, PLA 302 Hospital, Beijing, People's Republic of China; 7 Department of Orthopaedics, Tangdu Hospital, The Fourth Military Medical University, Xi'an, People's Republic of China; University of South Carolina School of Medicine, United States of America

## Abstract

Postherpetic neuralgia (PHN), the most common complication of herpes zoster (HZ), plays a major role in decreased life quality of HZ patients. However, the neural mechanisms underlying PHN remain unclear. Here, using a PHN rat model at 2 weeks after varicella zoster virus infection, we found that spinal astrocytes were dramatically activated. The mechanical allodynia and spinal central sensitization were significantly attenuated by intrathecally injected L-α-aminoadipate (astrocytic specific inhibitor) whereas minocycline (microglial specific inhibitor) had no effect, which indicated that spinal astrocyte but not microglia contributed to the chronic pain in PHN rat. Further study was taken to investigate the molecular mechanism of astrocyte-incudced allodynia in PHN rat at post-infection 2 weeks. Results showed that nitric oxide (NO) produced by inducible nitric oxide synthase mediated the development of spinal astrocytic activation, and activated astrocytes dramatically increased interleukin-1β expression which induced *N*-methyl-D-aspartic acid receptor (NMDAR) phosphorylation in spinal dorsal horn neurons to strengthen pain transmission. Taken together, these results suggest that spinal activated astrocytes may be one of the most important factors in the pathophysiology of PHN and “NO-Astrocyte-Cytokine-NMDAR-Neuron” pathway may be the detailed neural mechanisms underlying PHN. Thus, inhibiting spinal astrocytic activation may represent a novel therapeutic strategy for clinical management of PHN.

## Introduction

The most common complication of herpes zoster (HZ) is postherpetic neuralgia (PHN) which has been defined as severe pain occurring 1 month after rash onset and persisting for more than 3 months [1]. PHN is often prolonged, responds poorly to current analgesics such as anticonvulsants, tricyclic antidepressants, opioids and non-steroidal anti-inflammatory drugs (NSAIDS) [2]. PHN can result in impaired sleep, emotional distress and depression which patients may suffer for years [3]. Continued inflammation was observed in biopsy studies of some PHN patients, whereas PHN is resistant to NSAIDS, suggesting that inflammatory response per se is not sufficient to induce PHN [4], [5]. Recently, it has been pointed out that PHN shares some characteristics of neuropathic pain [6]. For example, up-regulation of some neuropeptide, calcium channel and sodium channel has been observed in dorsal root ganglia of PHN rat model. Besides, systemic treatment with gabapentin or the sodium channel blockers could to some extent reverse PHN [7]. However, there is still no ideal explanation on the neural mechanisms underlying PHN.

According to classic pain research, the pain pathway has been assumed to be composed &&entirely of neuronal synaptic transmission. It has been widely accepted that activation of *N*-methyl-D-aspartate receptor (NMDAR) in spinal postsynaptic neurons plays an important role in neuropathic pain, and NMDAR antagonists are potential analgesics [8], [9]. NMDAR activation is mainly manifested by NR1 subunit phosphorylation, which is proposed to be involved in spinal central sensitization [10], [11]. However, recent studies have shown that spinal cord glia and proinflammatory cytokines, such as interleukin (IL)-1β, are also identified to be strongly involved in the creation and maintenance of diverse exaggerated neuropathic pain states [12], [13]. After inflammation or nerve injury, spinal glial cells can be activated by excessively produced nitric oxide (NO), and then synthesize and release IL-1β that modulate neuronal activity [14], [15]. Several recent studies showed that IL-1β may bind to its endogenous receptor to induce the phosphorylation of NR1 subunit of NMDAR to strengthen painful signal transmission [16], [17]. However, there is still no related report on the involvement of spinal glial cells in PHN.

Investigation of the neural mechanisms underlying PHN has been facilitated by the introduction of an *in vivo* rat model of varicella zoster virus (VZV) persistent infection [18], [19]. Recent studies indicated that this rat model could ideally mimic the chronic pain states that occur in PHN patients [20], [21]. In this study, we investigated the role of spinal glia in the pathophysiology of PHN by using this PHN model. L-α-aminoadipate (LAA) and minocycline were used to inactivate astrocyte and microglia, respectively, to identify the roles of spinal glial cells in the development of PHN. LAA was used to inhibit astrocytes based on the fact that its role of a specific astrocytic toxin [22]. In addition, intrathecal treatment with inhibitors of nitric oxide synthase (NOS) or scavenger of NO was performed to test whether NO mediate the development of glial activation. The mediating role of inflammatory cytokine on NMDAR activation was also investigated.

## Materials and Methods

### Animals

Adult male Wistar rats, weighing 200–250 g, were used. Rats were housed under standard conditions. All procedures of our experiments were approved by the Committee of Animal Use for Research and Education of the Fourth Military Medical University (Xi'an, PR China), and all efforts were made to minimize the number of animals used and their suffering [23]. (Permit Number: fmmu-10-6688).

### Varicella zoster virus (VZV) infection

We utilized a previously reported PHN model of latent VZV infection [18], [20], [21]. VZV (VR-568 mycoplasma free strain obtained from ATCC, VA, USA) was propagated on CV-1 cells (African green monkey kidney fibroblast cells) and harvested in 0.0l M phosphate-buffered saline (PBS, pH 7.4) when the cells exhibited an 80% cytopathic effect. Rats were anaesthetised with halothane and the plantar surface of the right hindpaw was injected with 50 µl of inoculum containing 6×10^6^ VZV-infected cells (VZV group). The contralateral hindpaw was without infection. Control rats were injected with uninfected CV-1 cells (Mock infected group) or PBS (Naïve group) and housed separately from VZV group. Pain behavioural tests were performed prior to infection to obtain a baseline and then at specific time points post-infection.

### Antibodies

Primary antibodies: mouse anti-GFAP IgG (astrocytic marker; Chemicon, Temecula, CA, USA), mouse anti-NeuN IgG (neuronal marker; Chemicon), mouse anti-OX42 IgG (microglial marker; Chemicon), rabbit anti-IL-1β IgG (Endogen, Rockford, IL, USA), rabbit anti-P-ser896 NR1 IgG (Millipore, Bedford, MA, USA), rabbit anti-iNOS IgG (Calbiochem, San Diego, CA, USA), rabbit anti-nNOS IgG (Calbiochem) and rat anti-IL-1RI IgG (Santa Cruz Biotechnology; Santa Cruz, CA, USA). Secondary antibodies: FITC or Cy3-labeled donkey anti-mouse IgG (Chemicon), FITC or Cy3-labeled donkey anti-rabbit IgG (Chemicon), FITC or Cy3-labeled donkey anti-rat IgG (Chemicon).

### Drugs

Chemicals and their sources were as follows: L-α-aminoadipate (LAA, astrocytic specific inhibitor; Sigma, St. Louis, MO, USA), minocycline (microglial specific inhibitor; Sigma), 2-(4-carboxyphenyl)-4,4,5,5-tetramethylimidazoline-1-oxyl-3-oxide (PTIO, scavenger of NO; Sigma), L-N6-(I-iminoethyl)-lysine hydrochloride (L-NIL, inhibitor of iNOS; Sigma), 7-Nitroindazole (7-NINA, inhibitor of nNOS; Sigma), pentoxifylline (cytokine inhibitor; Polfilin, Polfarma, Poland), interleukin-1 receptor antagonist (IL-1ra; Amgen, Thousand Oaks, CA, USA), 5-aminophosphonovaleric acid (AP5, NMDA receptor antagonist; Sigma) and (5*R*,10*S*)-(+)-5-methyl-10,11-dihydro-5*H*-dibenzo[*a*,*d*]cyclo-hepten-5,10-imine hydrogen maleate (MK-801, non-competitive NMDA receptor antagonist; Sigma), valaciclovir (Glaxo Wellcome, USA).

### Experimental design

**Figure 1:**
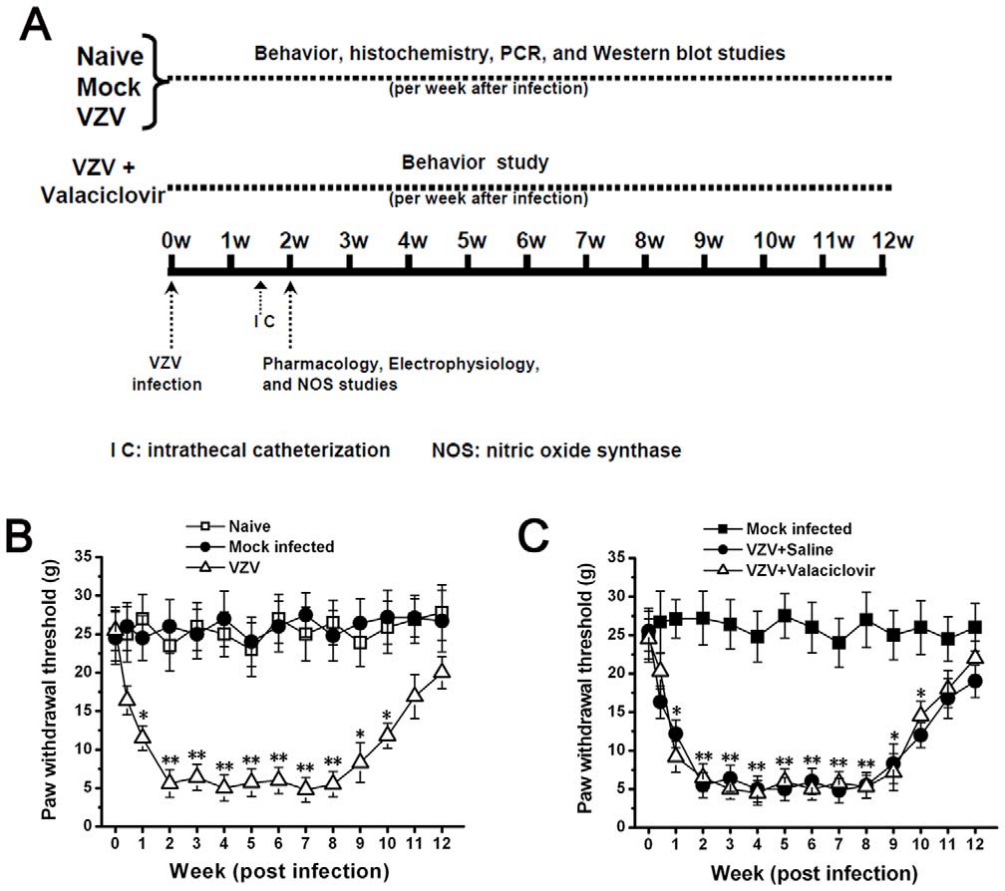
Experimental procedures in this study (A) and mechanical allodynia in VZV infected rats (B and C). (A) The timeline represents the period during which behavior, histochemistry, PCR and Western blot studies were performed per week after VZV infection. Intrathecal catheterization was performed on rats and followed by 3-day recovery. The pharmacology, electrophysiology, and NOS studies were conducted at post-infection 2 weeks when the mechanical allodynia reached the highest level. (B) Compared with Naive rats and Mock infected rats, the paw withdrawal threshold of VZV infected rats was significantly decreased. * *P*<0.05, ** *P*<0.01 vs. Naive rats and Mock infected rats. (C) Systemic treatment with antiviral agent valaciclovir had no effect on the development of mechanical allodynia. * *P*<0.05, ** *P*<0.01 vs. Mock infected rats. All data were calculated as mean ± SEM (n = 10/group/week).

In the first series of experiments, rats were divided into Naive group, Mock infected group and VZV group. At post-infection different time points (per week after VZV infection), pain behavior, immunostaining, Western blot and real-time RT-PCR studies were performed in each group. Also, some VZV infected rats received antiviral treatment (Valaciclovir, 50 mg/kg/day from post-infection day 1 to day 10, i.p) and pain behavior was detected per week after infection. (n = 10/group; Fig. 1A).

In the second series of experiments, saline, LAA, minocycline, PTIO, L-NIL, 7-NINA, pentoxifylline, IL-1ra, AP5 or MK-801 was injected intrathecally in VZV-infected rats at post-infection 2 weeks. After injection, pain behavior was immediately measured (n = 10/group).

In the third series of experiments, rats at post-infection 2 weeks were used. LAA or minocycline was injected intrathecally, and one hour later a total of 30 wide dynamic range (WDR) neurons were recorded in each group (naive group, mock infected group, VZV group, VZV+LAA group and VZV+minocycline group; n = 30/group).

In the fourth series of experiments, rats at post-infection 2 weeks were used. Firstly, the L5 dorsal root ganglia and spinal cord segments were harvested for Western blot analysis of iNOS or nNOS in Naive group, Mock infected group and VZV group (n = 10/group). Secondly, double-labeling immunofluorescence of iNOS with NeuN , GFAP or OX42 was performed in dorsal root ganglia or spinal cord sections (n = 10/group). Thirdly, PTIO, L-NIL or 7-NINA was injected intrathecally, and one hour later the spinal cords of VZV-infected rats were harvested for Western blot analysis of GFAP (n = 10/group).

In the fifth series of experiments, LAA was injected intrathecally in VZV-infected rats at post-infection 2 weeks, and one hour later spinal cords were harvested for Western blot analysis of IL-1β (n = 10). Double-labeling immunofluorescence of IL-1β with GFAP or OX42 was performed in the L5 spinal cord sections.

In the sixth series of experiments, LAA, pentoxifylline or IL-1ra was injected intrathecally in VZV-infected rats at post-infection 2 weeks, and one hour later the spinal cords were harvested for Western blot analysis of P-NR1 (n = 10/group). Double-labeling immunofluorescence of IL-1RI and P-NR1 was performed in the L5 spinal cord sections.

### Intrathecal catheter insertion and drug administration

A polyethylene-10 catheter (Becton-Dickinson, Sparks, MD, USA) was intrathecally inserted according to a previous method [24]. The rats were allowed to recover for 3 days. Only the rats judged as neurologically normal were used for the subsequent drug administration. The dosage of each drug used in the present study was according to the previous reports [25], [26], [27], [28], [29], [30]. Treatment group received intrathecal injection of LAA (100 nmol) [25], minocycline (100 µg) [26], L-NIL (1.1 µmol) [27], PTIO (30 µg) [28], 7-NINA (20 µg) [27], AP5 (40 pmol) [27], MK-801 (100 pmol) [27], pentoxifylline (150 nmol) [29] or IL-1ra (100 µg) [30], while the same volume of normal saline was injected in control group. Each drug was dissolved in 10 µl of saline and injected intrathecally by the way of a single acute administration.

### Pain behavioral test

The rats were adapted to the testing situation for at least 15 min. The observers of the behaviors were blind to the treatment of the rats. As previously described [31], a set of von Frey monofilaments (Stoelting, Chicago, IL, USA) was used to test the mechanical withdrawal threshold of the hindpaws. The monofilaments were applied with increasing force until the rats withdrew the paw. The threshold was taken as the lowest force that evoked a brisk withdrawal response. To observe how different drug treatments affected the allodynia, behavioral tests were performed 12 h before the drugs administration to provide baseline scores. After intrathecal drug injection, mechanical withdrawal threshold was measured for every 10 min.

### Immunofluorescence histochemical staining

#### Tissue preparation

Rats were anesthetized and perfused transcardially with paraformaldehyde. The L5 dorsal root ganglia (DRG) and the L5 spinal cord were removed and transferred into 30% sucrose in 0.1 M phosphate buffer (PB, pH 7.4) for cryoprotection. Spinal cord was cut into 30 µm thick sections and collected in 0.01 M phosphate-buffered saline (PBS, pH 7.4). The DRG was cut into longitudinal sections measuring 10 µm thick and mounted onto gelatin-coated glass slides. 10 representative tissue sections from each rat were selected (10 rats/group). After being blocked with 10% normal goat serum (NGS), sections were incubated with corresponding antibodies.

#### Single immunofluorescence

After washed in PBS containing 0.3% Triton X-100 (PBS-X, pH 7.4), the spinal cord sections were incubated sequentially with: (1) mouse anti-GFAP IgG (1∶500) or mouse anti-OX42 IgG (1∶200) in 0.0l M PBS containing 5% (v/v) normal donkey serum (NDS), 0.3% (v/v) Triton X-100, 0.05% (w/v) NaN_3_ and 0.25% (w/v) carrageenan (PBS-NDS, pH 7.4) for 48 h at 4°C; (2) FITC-labeled donkey anti-mouse IgG (1∶200) in PBS-NDS for 12 h at 4°C.

#### Double immunofluorescence

Rabbit anti-IL-1β IgG (1∶300), rabbit anti-P-ser896 NR1 IgG (1∶500), rat anti-IL-1RI IgG (1∶600) and rabbit anti-iNOS IgG (1∶400) were respectively double labelled with mouse anti-GFAP IgG (1∶500), mouse anti-NeuN IgG (1∶1000) or mouse anti-OX42 IgG (1∶200) in the spinal cord sections; rabbit anti-P-ser896 NR1 IgG (1∶500) was double labelled with rat anti-IL-1RI IgG (1∶600) in the spinal cord sections; rabbit anti-iNOS IgG (1∶400) and rabbit anti-nNOS IgG (1∶400) were respectively double labelled with mouse anti-GFAP IgG (1∶500), mouse anti-NeuN IgG (1∶1000) or mouse anti-OX42 IgG (1∶200) in the DRG sections.

Between each step, the sections were washed with PBS for three times. After staining, the sections were coverslipped with a mixture of 50% (v/v) glycerin and 2.5% (w/v) triethylene diamine (anti-fading agent) in PBS, and observed with a confocal laser scanning microscope (Olympus FV1000, Tokyo, Japan) under appropriate filters for green-emitting FITC (excitation 490 nm; emission 520 nm) and for red-emitting Cy3 (excitation 552 nm; emission 565 nm).

### Western blot analysis

Rats were anesthetized and the dorsal halves of the spinal cord innervated by the L5 dorsal roots were rapidly removed. The collected tissue was mechanically homogenized and centrifuged. The supernatant was collected and stored at −80°C. Protein concentrations of the supernatant were determined using the BCA Protein Assay Kit (Pierce, Rockford, IL, USA). Proteins of interest were separated by SDS-PAGE electrophoresis (20 µg of total protein per well), and transferred onto nitrocellulose membranes. The membranes were placed in a blocking solution (TBS with 0.02% Tween and 5% non-fat dry milk powder) for 1 h, and incubated overnight with mouse anti-GFAP IgG (1∶500), mouse anti-OX42 IgG (1∶200), rabbit anti-iNOS IgG (1∶400), rabbit anti-nNOS IgG (1∶400), rabbit anti-IL-1β IgG (1∶300) or rabbit anti-P-ser896 NR1 IgG (1∶500). After washing, the membranes were incubated in peroxidase-conjugated secondary antibody (1∶1000; Santa Cruz) for 1 h, and then the membranes were detected by the enhanced chemiluminescence detection method (Amersham Pharmacia Biotech Inc., Piscataway, NJ, USA). The densities of protein blots were analyzed by using Labworks Software (Ultra-Violet Products Ltd., Cambridge, UK) and normalized to β-actin levels.

### Real-time reverse transcription polymerase chain reaction (RT-PCR)

Rats were anesthetized and L5 of the spinal dorsal horn was rapidly harvested. Total RNA was extracted with Trizol (GIBCO/BRL Life Technologies Inc., Grand Island, NY, USA), an RNA isolation reagent. cDNA was synthesized with oligo (dT)_12–18_ using Superscript^TM^ Reverse Transcriptase for RT-PCR (Invitrogen, Carlsbad, CA, USA). The primers used were presented in Table 1. GAPDH was served as an endogenous internal standard control. The PCR reactions were carried out using 2 µl of cDNA, primers specific to the gene of interest (Table 1) and the SYBR® Premix Ex Taq™ (Takara, Tokyo, Japan) in 20 µl reactions. Levels of PCR product were measured using SYBR Green fluorescence collected during real time PCR in a detection system (Applied Biosystems 7300, Foster City, CA, USA). Melting point analyses were performed for each reaction to confirm single amplified products. Target RNA sequence quantities were estimated from the threshold amplification cycle number (C_t_) using Sequence Detection System software (Applied Biosystems). Expression was normalized to GAPDH.

**Table 1:** Primers and Taqman probe sequence for the rat genes characterized in this experiment.

Genes	Primers	
GFAP	Forward primer	5′ TGGCCACCAGTAACATGCAA 3′
	Reverse primer	5′ CAGTTGGCGGCGATAGTCAT 3′
	Taqman probe	5′ CAGACGTTGCTTCCCGCAACGC 3′
OX42	Forward primer	5′ CTGCCTCAGGGATCCGTAAAG 3′
	Reverse primer	5′ CCTCTGCCTCAGGAATGACATC 3′
	Taqman probe	5′ CCCGGGACAATGCCGCGAA 3′
IL-1β	Forward primer	5′ GCAAACAGGTCGGCGTCTT 3′
	Reverse primer	5′ TGCGCAGCGCTAAAACTTG 3′
	Taqman probe	5′ TGATCTCGGCCCTCTGTCCGCA 3′
GAPDH	Forward primer	5′ CCCCCAATGTATCCGTTGTG 3′
	Reverse primer	5′ TAGCCCAGGATGCCCTTTAGT 3′
	Taqman probe	5′ TGCCGCCTGGAGAAACCTGCC 3′

### Electrophysiological testing

The rats were anesthetized and artificially ventilated. Core body temperature was monitored and maintained at 37.5±0.5°C. A laminectomy was performed from the T13 to L2 vertebrae to expose the lumbosacral enlargement of the spinal cord. Extracellular single unit recordings were made from L5 spinal dorsal horn ipsilateral to VZV infection with glass capillary microelectrodes (10–15 MΩ filled with 0.5 M sodium acetate). The dorsal horn neurons were identified as WDR units on the basis of their characteristic responses: (1) having a receptive field consisting of a small low threshold center and a large high threshold surround ipsilateral to the recording site; (2) responding with an increasing firing rate to brush, pressure and noxious pinch applied to the low threshold center; (3) showing no apparent accommodation when continuous noxious stimulation was applied. After successful identification of a single WDR unit, the unit responsiveness to 10 s mechanical stimuli was recorded. The spike trains were monitored with a memory oscilloscope and the numbers of neuronal firing were simultaneously recorded and saved on a computer via an A/D converter following spike discriminator and counter.

### Data analysis

The results were presented as mean ± SEM. Statistical analysis of the data was carried out with a one-way analysis of variance (ANOVA) followed by Bonferroni post hoc analysis. Comparisons between two means were performed by a Student's T-test. A Pearson correlation was used to determine the correlation between the intensity of GFAP (Western blot) and pain behavioral performance. Significance level was set at P<0.05. The statistics software used was SPSS 12.0 for Windows.

## Results

### VZV infection induced mechanical allodynia which was resistant to antiviral therapy

No difference in paw withdrawal threshold was observed between naive rats (25±3.5 g) and mock infected rats (24.5±3.4 g), and the paw withdrawal threshold of these two groups maintained at basal level through the period tested. Compared to naive rats and mock infected rats, the paw withdrawal threshold of VZV infected rats significantly decreased at post-infection 1 week (P1) (11.5±1.6 g), reached the lowest value at P2 (5.6±1.8 g), and thereafter maintained at low level till P8 (n = 10/group; *P*<0.05). After P8, the paw withdrawal threshold of VZV infected rats gradually increased to the basal level at P12 (Fig. 1B). On the other hand, some VZV infected rats were treated with antiviral agent valaciclovir. However, valaciclovir treatment had no effect on the development of mechanical allodynia (decreased paw withdrawal threshold), which suggested that continued virus infection was not required for VZV induced allodynia (Fig. 1C).

### Spinal astrocytic activation contributed to mechanical allodynia

**Figure 2:**
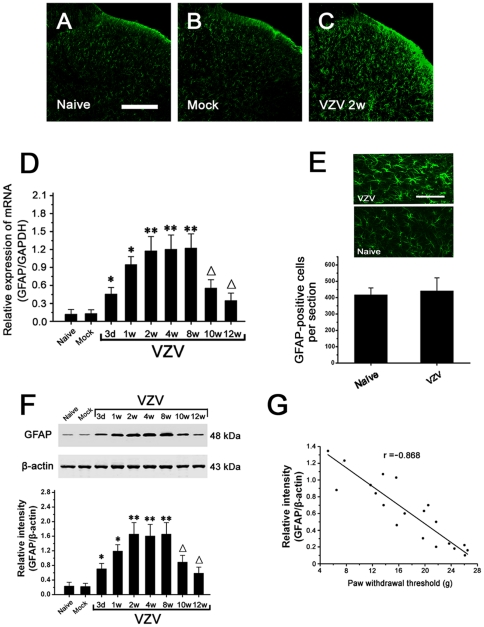
Spinal astrocytes were activated in VZV infected rats, which was significantly correlated to mechanical allodynia. (A–C) Compared with Naive rats and Mock infected rats, GFAP-like immunoreactivity (-LI) in spinal dorsal horn of VZV infected rats was significantly increased. Bar = 200 µm. (D) Real-time RT-PCR showed that spinal mRNA expression of GFAP was significantly upregulated in VZV infected rats compared to naive rats and mock infected rats. (E) With regard to the total number of GFAP positive cells, there was no difference between Naïve rats and VZV infected rats. Bar = 10 µm. (F) Compared to Naive rats and Mock infected rats, Western blot analysis showed that spinal GFAP expression was significantly increased in VZV infected rats. (G) The expression level of GFAP was found to be significantly correlated to the paw withdrawal threshold in VZV infected rats (*P*<0.001, r = −0.868). All data were calculated as mean ± SEM (n = 10/group/week). * *P*<0.05, ** *P*<0.01 vs. Naive rats and Mock infected rats; Δ*P*<0.05 vs. post-infection 2 weeks rats.

Immunohistochemistry indicated that compared to naive rats and mock infected rats, GFAP staining was significantly increased in the spinal cord of VZV infected rats at post-infection 1 week (P1), peaked at P2, and thereafter maintained at high level till P8. Staining of GFAP appeared to be enhanced throughout the spinal dorsal horn. (Fig. 2A–C). Using real-time RT-PCR, it was found that the spinal mRNA expression of GFAP was significantly upregulated in VZV infected rats compared to naive rats and mock infected rats (Fig. 2D). Activated astrocytes had hypertrophied cell bodies and thickened processes with enhanced GFAP-immunoreactivity in VZV infected rats. However, with regard to the total number of GFAP positive cells, there was no difference between Naïve rats and VZV infected rats (Fig. 2E). On the other hand, we found that BrdU immunoreactivity was undetectable in both Naïve rats and VZV infected rats in BrdU incorporation analysis, which indicated that there was no proliferation of astrocytes in the spinal cord of VZV infected rat (data not shown). Using Western blot, we detected that compared to naive rats (0.22±0.04) and mock infected rats (0.2±0.03), GFAP expression was significantly increased in VZV infected rats at post-infection day 3 (0.68±0.15). GFAP upregulation peaked at P2 (1.62±0.3) and thereafter persisted at high level till P8 (n = 10/group; *P*<0.05). After P8, GFAP expression gradually decreased to the basal level at P12 (Fig. 2F). Furthermore, the expression level of GFAP was found to be significantly correlated to the paw withdrawal threshold in VZV infected group (P<0.001, r = −0.868) (Fig. 2G).

**Figure 3:**
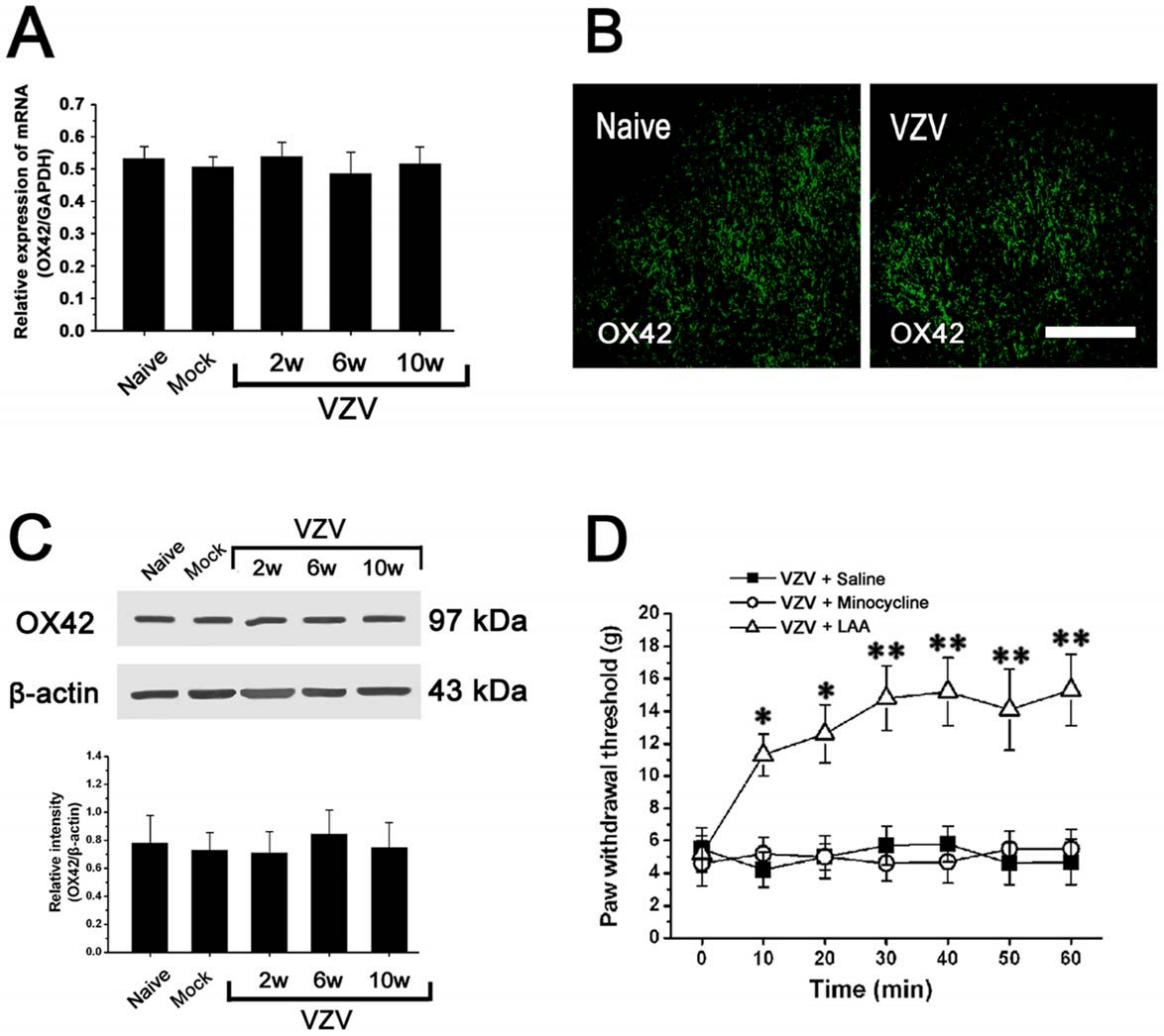
Spinal microglia was not activated in VZV infected rats, astrocytic specific inhibitor LAA but not microglial specific inhibitor minocycline could attenuate mechanical allodynia. (A) No significant difference in OX42 mRNA expression in spinal cord was observed among naive rats, mock infected rats and VZV infected rats. (B) With regard to OX42-like immunoreactivity (-LI) in spinal dorsal horn, there was no difference between Naïve rats and VZV infected rats. Bar = 200 µm. (C) With regard to OX42 expression in spinal cord, there was no difference among Naive rats, Mock infected rats and VZV infected rats. In VZV infected rats, OX42 expression was unchanged through the period tested. (D) Intrathecal injection of LAA significantly attenuated the allodynia. However, minocycline did not influence the allodynia. All data were calculated as mean ± SEM (n = 10/group). * *P*<0.05, ** *P*<0.01 vs. VZV+Saline group or VZV+minocycline group in D.

No significant difference in OX42 mRNA expression was observed among naive rats, mock infected rats and VZV infected rats (Fig. 3A). With regard to OX42-like immunoreactivity (-LI) in spinal dorsal horn, there was no difference between Naïve rats and VZV infected rats (Fig. 3B). With regard to OX42 expression in spinal cord, Western blot showed that there was no difference between naive rats, mock infected rats and VZV infected rats at post-infection any week. In all the mice, OX42 expression was unchanged through the period tested (Fig. 3C). We injected LAA or minocycline intrathecally and observed their effects on mechanical allodynia in VZV infected rats (P2). The astrocytic specific toxin LAA significantly attenuated the allodynia. However, the microglial specific inhibitor minocycline did not influence mechanical allodynia (Fig. 3D).

### Spinal astrocytic activation contributed to spinal central sensitization in VZV infected rat

**Figure 4:**
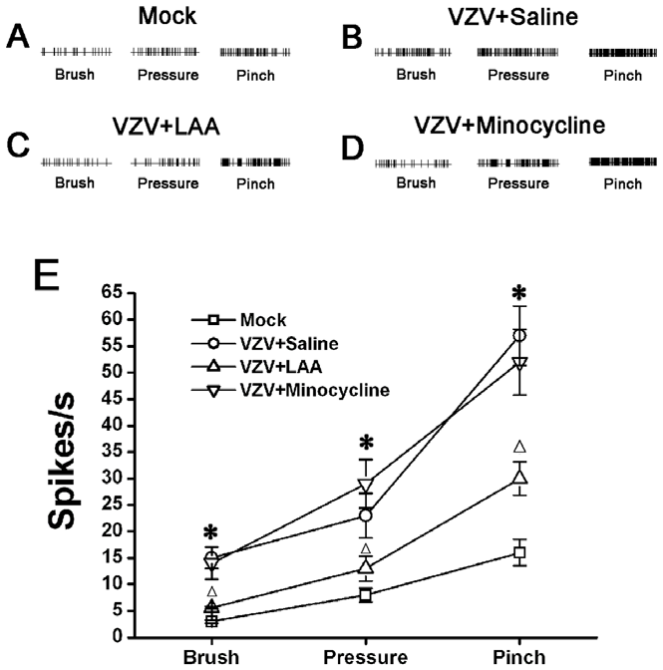
Astrocytic activation contributed to spinal central sensitization in VZV infected rats. Comparative recordings of responsiveness of spinal dorsal horn wide dynamic range (WDR) neurons to mechanical (brush, pressure and pinch) stimuli in Mock infected group (A), VZV infected group (B), VZV+LAA group (C) and VZV+minocycline group (D). (A–D) The responsiveness of WDR neurons was gradedly increased with the increase in mechanical intensity (brush, pressure and pinch). (E) Compared to Mock infected rats, the responsiveness of WDR neurons was significantly enhanced with a distinct leftward shift of the stimulus-response functional curve in VZV infected rats. The astrocytic specific toxin LAA significantly attenuated the increased responsiveness. However, the microglial specific inhibitor minocycline did not have any effect. * *P*<0.05 vs. Mock infected rats; Δ*P*<0.05 vs. VZV infected rats.

The responsiveness of WDR neurons was gradedly increased with the increase in mechanical intensity (brush, pressure and pinch) (Fig. 4A–D). The stimulus-response functional curves for mechanical sensitivity of the spinal dorsal horn WDR neurons are shown in each group (Fig. 4E). Compared to mock infected rats, the responsiveness of WDR neurons was significantly enhanced with a distinct leftward shift of the stimulus-response functional curve in VZV infected rats, which indicated that spinal central sensitization occurred in VZV infected rat (Fig. 4E). We injected LAA or minocycline intrathecally and observed their effects on increased responsiveness of WDR neurons in VZV infected rats (P2). The astrocytic specific toxin LAA significantly attenuated the increased responsiveness. However, the microglial specific inhibitor minocycline did not have any effect (Fig. 4E).

### Spinal astrocytic activation depended on activation of neuronal iNOS

**Figure 5:**
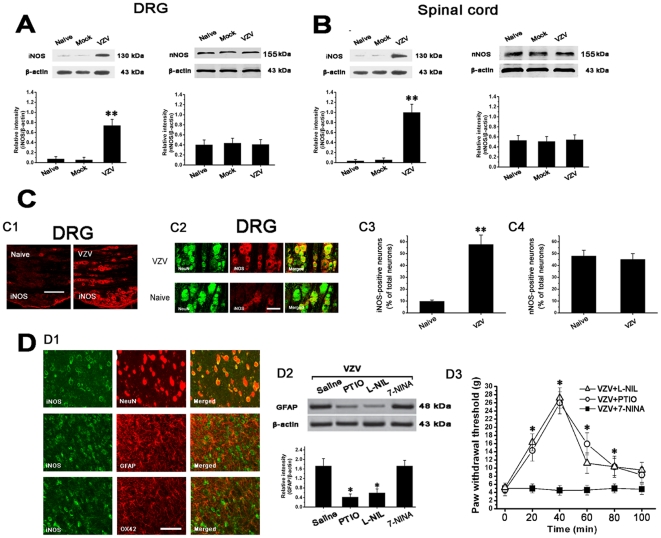
VZV infection induced spinal astrocytic activation depended on activation of iNOS. (A and B) Compared to Naive rats and Mock infected rats, iNOS expression was significantly increased in dorsal root ganglion (DRG) and spinal cord of VZV infected rats. With regard to nNOS expression in DRG and spinal cord, there was no difference among Naive rats, Mock infected rats and VZV infected rats. (C1–C4) The number of iNOS-immunopositive but not nNOS-immunopositive DRG neurons in VZV infected rats was significantly increased compared to Naive rats. Bars = 100 µm (C1) and 20 µm (C2). (D1) iNOS-immunoreactivity was localized in NeuN-immunopositive cells but not in GFAP-immunopositive cells or OX42-immunopositive cells in spinal cord of VZV infected rats. Bar = 50 µm. (D2) Intrathecal treatment with L-NIL (selective inhibitor of iNOS) or PTIO (scavenger of NO) significantly reduced GFAP overexpression in spinal cord of VZV infected rats. However, intrathecal treatment with 7- NINA (selective inhibitor of nNOS) had no effect on GFAP overexpression in VZV infected rats. (D3) Intrathecal treatment with L-NIL or PTIO exerted significant analgesic effect in VZV infected rats. However, intrathecal treatment with 7-NINA had no effect on pain behavior in VZV infected rats. All data were calculated as mean ± SEM (n = 10/group). ** *P*<0.01 vs. Naive rats and Mock infected rats in A–C. * *P*<0.05 vs. saline (vehicle) treated VZV infected rats in D2.

Compared to naive rats and mock infected rats, iNOS expression was significantly increased in DRG and spinal cord of VZV infected rats (P2). However, with regard to nNOS expression in DRG and spinal cord, there was no difference among Naive rats, Mock infected rats and VZV infected rats (Fig. 5A and B). Also, the number of iNOS-immunopositive but not nNOS-immunopositive DRG neurons in VZV infected rats was significantly increased when compared to naive rats (Fig. 5C1–C4).

At P2 in VZV infected rats, double immunofluorescent staining of spinal cord showed that iNOS-immunoreactivity was only localized in NeuN-immunopositive cells but not in GFAP-immunopositive cells or OX42-immunopositive cells (Fig. 5D1). In addition, intrathecal treatment with L-NIL (inhibitor of iNOS) or PTIO (scavenger of NO) significantly reduced GFAP overexpression in VZV infected rats. However, intrathecal treatment with 7-NINA (selective inhibitor of nNOS) had no effect on GFAP overexpression in VZV infected rats (Fig. 5D2). Furthermore, intrathecal treatment with L-NIL or PTIO exerted significant analgesic effect in VZV infected rats. However, intrathecal treatment with 7-NINA had no effect on pain behavior in VZV infected rats (Fig. 5D3). All these data indicated that VZV infection-induced activation of neuronal iNOS may mediate the development of spinal astrocytic activation and allodynia in VZV infected rats.

### Spinal astrocytes dramatically increased the expression of IL-1β which was related to mechanical allodynia

**Figure 6:**
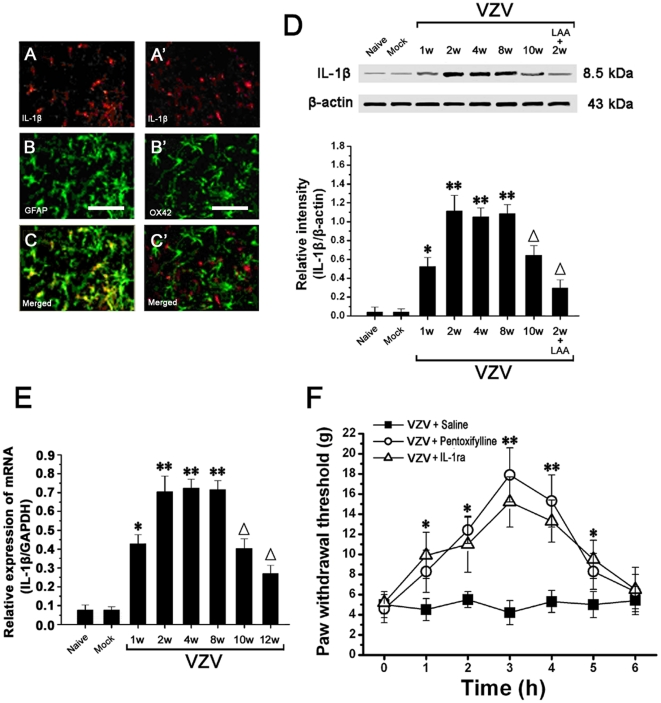
IL-1β overexpression in spinal cord was related to mechanical allodynia in VZV infected rats, and activated astrocytes were the only source of IL-1β. (A–C′) Double immunofluorescent staining showed that IL-1β-immunoreactivity was localized in GFAP-immunopositive cells but not OX42-immunopositive cells in spinal cord of VZV infected rats. Bars = 20 µm. (D) IL-1β expression was significantly increased in spinal cord of VZV infected rats compared to Naive rats and Mock infected rats. Intrathecal treatment with LAA (astrocytic specific toxin) significantly reduced IL-1β overexpression in VZV infected rats. (E) Using real-time RT-PCR, it was found that the spinal mRNA expression of IL-1β was significantly upregulated in VZV infected rats compared to naive rats and mock infected rats. (F) Intrathecal injection of Pentoxifylline (cytokine inhibitor) or IL-1ra (interleukin-1 receptor antagonist) could significantly attenuated the allodynia. All data were calculated as mean ± SEM (n = 10/group). * *P*<0.05, ** *P*<0.01 vs. Naive rats and Mock infected rats; Δ*P*<0.05 vs. post-infection 2 weeks rats in D and E. * *P*<0.05, ** *P*<0.01 vs. saline (vehicle) treated VZV infected rats in F.

At post-infection 2 week (P2) in VZV infected rats, double immunofluorescent staining of spinal cord showed that IL-1β-immunoreactivity was only localized in GFAP-immunopositive cells but not in OX42-immunopositive cells (Fig. 6A–C′). Western blot analysis showed that compared to naive rats (0.04±0.006) and mock infected rats (0.043±0.005), IL-1β expression was significantly increased in VZV infected rats at post-infection 1 week (P1) (0.5±0.11). IL-1β upregulation peaked at P2 (1.1±0.16), and thereafter persisted at high level till P8 (n = 10/group; *P*<0.05). After P8, IL-1β expression gradually decreased to the basal level at P12 (Fig. 6D). Thus, the time course of IL-1β expression was similar to that of GFAP expression. At P2 in VZV infected rats, intrathecally administered LAA could significantly down-regulate IL-1β overexpression (0.28±0.07) (Fig. 6D). Using real-time RT-PCR, it was found that the spinal mRNA expression of IL-1β was significantly upregulated in VZV infected rats compared to naive rats and mock infected rats (Fig. 6E). At P2 in VZV infected rats, we injected pentoxifylline (cytokine inhibitor) or IL-1ra (interleukin-1 receptor antagonist) intrathecally and observed their effects on mechanical allodynia in VZV infected rats. Both pentoxifylline and IL-1ra could significantly attenuated the allodynia (Fig. 6F). In addition, we found that intrathecal treatment with pentoxifylline or IL-1ra had no effect on GFAP overexpression in VZV infected rats (data not shown), which indicated that IL-1β may had no effect on spinal astrocytes in VZV-infected rats.

### IL-1β induced NMDA receptor phosphorylation which contributed to mechanical allodynia

**Figure 7:**
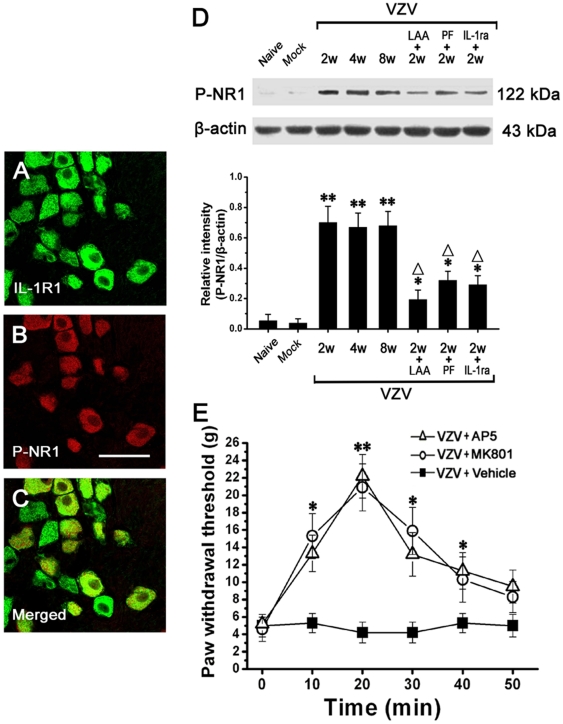
IL-1β released from astrocyte induced NMDA receptor phosphorylation in spinal dorsal horn neurons in VZV infected rats, which was related to mechanical allodynia. (A–C) Double immunofluorescent staining showed that P-NR1-immunoreactivity and IL-1RI-immunoreactivity were totally double-labeled in spinal dorsal horn of VZV infected rats. Bar = 50 µm. (D) The phosphorylation of NR1 was significantly increased in VZV infected rats compared to Naive rats and Mock infected rats. Intrathecal treatment with LAA (astrocytic specific toxin), PF (cytokine inhibitor) or IL-1ra (interleukin-1 receptor antagonist) could significantly reduce the phosphorylation of NR1 in VZV infected rats. (E) Intrathecal injection of AP5 (NMDA receptor antagonist) or MK-801 (non-competitive NMDA receptor antagonist) could significantly attenuated the allodynia. All data were calculated as mean ± SEM (n = 10/group). * *P*<0.05, ** *P*<0.01 vs. Naive rats and Mock infected rats; Δ*P*<0.05 vs. post-infection 2 weeks rats in D. * *P*<0.05, ** *P*<0.01 vs. saline (vehicle) treated VZV infected rats in E.

At P2 in VZV infected rats, double immunofluorescent staining of spinal cord showed that P-NR1-immunoreactivity and IL-1RI-immunoreactivity were only localized in NeuN-immunopositive cells, and P-NR1-immunoreactivity and IL-1RI-immunoreactivity were totally double-labeled (Fig. 7A–C).

Western blot analysis showed that compared to naive rats (0.05±0.009) and mock infected rats (0.04±0.006), the phosphorylation of NR1 was significantly increased in VZV infected rats at P1 (0.38±0.06). The phosphorylation of NR1 peaked at P2 (0.68±0.12), and thereafter persisted at high level till P8 (n = 10/group; *P*<0.05). After P8, the phosphorylation of NR1 gradually decreased to the basal level at P12 (Fig. 7D). Thus, the time course of the level of P-NR1 was similar to that of IL-1β expression or GFAP expression. At P2 in VZV infected rats, intrathecally administered LAA, pentoxifylline or IL-1ra could significantly reverse infection induced phosphorylation of NR1 (Fig. 7D). At P2 in VZV infected rats, we injected AP5 (NMDA receptor antagonist) or MK-801 (non-competitive NMDA receptor antagonist) intrathecally and observed their effects on mechanical allodynia in VZV infected rats. Both AP5 and MK-801 could significantly attenuated the allodynia (Fig. 7E). Most importantly, we found that neither MK-801 nor AP5 could affect the motor performance of VZV infected rats in the rotarod test, which indicated that the antinociceptive effects of MK-801 or AP5 did not rely on impairments of motor function (data not shown). In addition, it was found that intrathecal treatment with MK-801 or AP5 had no effect on GFAP or IL-1β overexpression in VZV infected rats (data not shown).

## Discussion

The present study was the first time to provide evidence that spinal astrocytic activation contributed to mechanical allodynia in VZV-infected rats. The neural mechanism in astrocyte incudced allodynia may be that spinal activated astrocytes dramatically increased the expression of IL-1β which may induce NMDA receptor phosphorylation in spinal dorsal horn neurons to enhance neuronal activity and pain transmission.

### Spinal astrocyte but not microglia was activated in VZV infected rat, which contributed to experimental postherpetic neuralgia

PHN is characterized by the development of hyperalgesia (a facilitated behavioral response to painful stimuli), mechanical allodynia (the perception of innocuous stimuli as painful) and spontaneous pain, which are also features of other forms of neuropathic pain [32]. Although it is still unclear how latent VZV infection interacts with the nervous system to induce pain behavioural changes, the underlying pathophysiological mechanisms of PHN may be similar to other forms of neuropathic pain. Spinal cord glia (astrocyte and microglia) are now posited to be dynamically and powerfully involved in diverse exaggerated pain states [12], [13]. While cumulating evidence suggested the involvement of microglia in varius models of persistent pain [33], such as spinal nerve ligation induced pian [34], formalin induced inflammatory pain [35], spinal cord injury induced pian [36] and so on, emerging studies have suggested the critical role of spinal astrocytes in some pathological pain states, including neuropathic pain [25], inflammatory pain [37], visceral pain [38] and diabetic pain [39]. To identify the potential involvement of which subtype of glial cell (astrocyte or microglia) in PHN, we studied the expression of different glia activation markers in the spinal cord and the influence of different glial inhibitors on mechanical allodynia in VZV-infected rats. We found that GFAP (astrocytic activation marker) but not OX42 (microglial activation marker) was significantly increased in the spinal cord of VZV infected rat. The changing course of astrocytic activation was consistent with that of mechanical allodynia. Astrocytic specific inhibitor LAA but not microglial specific inhibitor minocycline significantly attenuated the allodynia, which elucidated that astrocytic activation but not microglial activation contributed to allodynia in VZV infected rat. To the best of our knowledge, we are the first to report that this glia cell (astrocyte) plays an important role in mechanical allodynia of VZV infected rat.

Similar to our finding, a recent study showed that spinal astrocytes but not microglia may be a crucial component of type 2 diabetes-induced neuropathic pain [39]. However, another recent study indicated that spinal microglia are more activated than astrocytes in the neuropathic pain of chronic constriction injury to the sciatic nerve [40]. Interestingly, a previous study showed that both spinal astrocytes and microglia were activated in the neuropathic pain of partial peripheral nerve injury [41]. All the above results indicated that spinal astrocytic activation and/or microglial activation play crucial roles in different pathological pain states. The discrepancy in the activation of glial cell subtypes (astrocyte or microglia) may be attributed to different pathogenetic mechanism within different models of neuropathic pain. Nevertheless, our promising findings regarding LAA-induced alleviation of mechanical allodynia in VZV infected rat suggest that pharmacological antagonism of astrocytic activation in spinal cord may offer a great advantage in the treatment of PHN.

### VZV infection-induced activation of neuronal iNOS may contribute to spinal astrocytic activation

A key factor in the neural plasticity underlying neuropathic pain is altered expression of neurotransmitters in sensory dorsal root ganglion (DRG) neurons [42]. Previous studies have shown that there was an increased expression of calcium channel, sodium channels, the neuropeptide and activating transcription factor-3 in DRG of VZV-infected rats [7]. In the present study, it was found that iNOS but not nNOS expression was significantly increased in DRG and spinal dorsal horn of VZV infected rats when compared with control rats. Also, the number of iNOS-immunopositive DRG neurons in VZV infected rats was significantly increased. iNOS could produce an excessive amount of NO, a molecule which is one of the important substances implicated in diverse exaggerated pain states [43]. NO has been shown to regulate expression of GFAP in primary cultured astrocytes [44], [45], [46]. Here, we hypothesized that VZV infection-induced activation of iNOS may be a key mechanism for the development of spinal astrocytic activation in VZV infected rat. Intrathecal treatment with L-NIL (inhibitor of iNOS) or PTIO (scavenger of NO) significantly reduced GFAP overexpression whereas 7-NINA (selective inhibitor of nNOS) had no effect, which suggested that NO originating from iNOS may function as an initiator of astrocyte activation in VZV-infected rats. All these results indicate that neuronal input and related chemical mediators may be essential for triggering spinal astrocytic activation after VZV infection.

### Spinal astrocytic activation induced central sensitization of spinal neurons in VZV infected rat

Spinal dorsal horn wide dynamic range (WDR) neurons are more plastic following tissue injury and are believed to be responsible for the spinally-organized nociceptive transmission [42]. In this study, electrophysiological recording from WDR neurons in the lumbar (L4–L5) spinal dorsal horn in VZV infected rat showed significantly increased responsiveness to stimuli, which indicated that spinal central sensitization occurred in VZV infected rat, and this central sensitization contributed to the allodynia seen in VZV infected rats. Most importantly, the astrocytic specific toxin LAA significantly attenuated the increased responsiveness of WDR neurons, which demonstrated that spinal astrocytes play an important role in the mediation of excessively increased activity of dorsal horn neurons. These results dictate a necessity for subsequent detailed studies of signal coupling between astrocytes and neurons in spinal dorsal horn of VZV infected rat.

### The neural mechanism of astrocyte induced allodynia may be “Astrocyte-Cytokine-NMDAR-neuron” pathway

Under pathological condition, activated astrocytes could release proinflammatory cytokines, of which IL-1β has become the research focus [12], [15]. In the present study, IL-1β expression was significantly increased in VZV infected rat compared to control rat, and IL-1β was selectively localized in astrocytes. In support of our findings, previous studies also reported selective localization of increased IL-1β in spinal astrocytes in bone cancer pain model [47] and Complete Freunds adjuvant-induced inflammatory pain model [37]. Furthermore, intrathecally injected pentoxifylline (cytokine inhibitor) or IL-1ra (interleukin-1 receptor antagonist) could significantly attenuated the allodynia in this study. Similar to our data, previous studies also showed that intrathecally application of IL-1ra could exert significant analgesic effect in Complete Freunds adjuvant-induced inflammatory pain model [48] and L5 spinal nerve transection-induced neuropathic pain model [49]. Most importantly, intrathecally administered astrocytic specific inhibitor LAA could significantly down-regulate IL-1β expression in the present study. Consistent with our data, a recent study also reported that IL-1β overexpression could be blocked by intrathecally administered LAA in a type 2 diabetes-induced neuropathic pain [39]. Taken together, all these results strongly suggest that activated astrocytes are the only source of IL-1β release which contributed to mechanical allodynia in VZV infected rat.

Recent studies indicate that spinal NMDA receptor activation mainly involves phosphorylation of the NR1 (a subunit of NMDA receptor), which is strongly correlated with induction and maintenance of persistent pain [8], [9]. Here, it was shown that spinal phosphorylation of the NR1 was significantly enhanced in VZV infected rat compared to control rat, and P-NR1-immunoreactivity were only localized in spinal neurons. In support of our findings, previous studies also reported significantly enhanced phosphorylation of the NR1 subunit in spinal neurons in peripheral heat stimulation-induced pain model [10], trigeminal inflammation pain model [50] and partial sciatic nerve ligation-induced neuropathic pain model [11]. Furthermore, intrathecally injected AP5 (NMDA receptor antagonist) or MK-801 (non-competitive NMDA receptor antagonist) could significantly attenuated the allodynia in VZV infected rat in this study, which indicated that NR1 phosphorylation induced activation of NMDA receptor surely contributed to PHN. Similar to our data, previous studies also showed that intrathecally application of NMDA receptor antagonists could exert antiallodynic effect in chronic constriction injury-induced neuropathic pain model [8] and peripheral heat stimulation-induced pain model [10].

IL-1R, a subfamily of the Toll/IL-1 receptor superfamily, is the endogenous binding receptor for IL-1β. IL-1R contains two subtypes: the type I IL-1R (IL-1RI) and the type II IL-1R. IL-1RI is a transmembrane molecule and responsible for IL-1 signaling and the type II IL-1R lacks an intracellular domain and is incapable of signal transduction [51]. The present study showed that IL-1RI-immunoreactivity were only localized in spinal neurons. Similar to our finding, a previous study also reported selective localization of IL-1RI in spinal dorsal horn neurons in a inflammatory pain model [52]. In the present study, the time course of the phosphorylation of the NR1 was similar to that of IL-1β or GFAP expression, and neuronal P-NR1-immunoreactivity and neuronal IL-1RI-immunoreactivity were totally double-labeled, which strongly supported a close interaction of IL-1β signaling with neuronal NMDA receptor. Thus, it was hypothesized that IL-1β mediated allodynia in VZV infected rat may be through binding its receptor IL-1RI on the neurons, and then possibly via intracellular signal transduction leading to the phosphorylation of NMDA receptor NR1 subunit. As expected, subsequent data showed that intrathecally administered LAA, pentoxifylline or IL-1ra each could blocked the phosphorylation of the NR1 in this study. Similar to our finding, two previous studies reported that IL-1ra could block the phosphorylation of the NR1 in inflammatiory pain models [30], [50]. Also, a recent study showed that phosphorylation of the NR1 could be alleviated by intrathecal application of LAA in spinal nerve ligation-induced neuropathic pain model [53]. Therefore, all the above results indicated that spinal activated astrocytes dramatically increased the expression of IL-1β which directly bind to its receptor IL-1RI to induce NMDA receptor phosphorylation in spinal dorsal horn neurons, and finally pain transmission was enhanced.

In summary, the present study suggestted that spinal activated astrocytes may be one of the most important etiological factors of PHN and “NO-Astrocyte-Cytokine-NMDAR-Neuron” pathway may be the detailed molecular mechanisms underlying astrocyte induced allodynia in PHN. These findings suggest that spinal astrocytic inhibition may hold a therapeutic promise in the treatment of postherpetic neuralgia.
